# Scientometric analysis of research productivity in clinical pharmacy and practice: a 12-year review at a Middle Eastern university

**DOI:** 10.1080/20523211.2025.2480154

**Published:** 2025-03-27

**Authors:** Ikram Zoukh, Myrna Tabet, Ahmed Awaisu

**Affiliations:** a Department of Clinical Pharmacy and Practice, College of Pharmacy, QU Health, Qatar University, Doha, Qatar; b Qatar University Library, Qatar University, Doha, Qatar

**Keywords:** Scientometric analysis, bibliometric analysis, higher education, clinical pharmacy, research productivity

## Abstract

**Background:**

Assessing faculty research productivity through scientometric analysis is crucial for academic institutions to promote and enhance research activities. Scientometric analysis guides institutional goals, identifies gaps and faculty contributions, and influences career development in academia. With advancements in the clinical pharmacy and practice field and Qatar's evolving research infrastructure, this study aimed to evaluate the research productivity and quality indicators of a clinical pharmacy and practice department at Qatar University through scientometric analysis.

**Methods:**

Research articles published between 2012 and 2024 were retrieved using Web of Science (WoS) via InCites, Scopus via SciVal, and Google Scholar. Metrics analysed included total publications, journal impact factor, Q ranking, subject area coverage, and collaborations.

**Results:**

Thirty-one faculty members produced 1,266 articles indexed in WoS, 1,270 in Scopus, and 1,737 in Google Scholar. The average annual publication rate was 5 articles per faculty member, resulting to an average of 41 publications per faculty member over their tenure. Active publication years accounted for 85% of faculty tenures. Furthermore, research output and productivity increased steadily, with the highest publication output observed between 2021 and 2023. Most publications appeared in journals with Q1 or Q2 rankings, with the *International Journal of Clinical Pharmacy* and *Research in Social and Administrative Pharmacy* being the most frequently targeted outlets. Publications covered diverse topics and subject areas, including pharmacy practice, health education, patient and medication safety, and chronic disease management. Database comparisons revealed differences in retrieval rates, journal impact indicators, and Q-rankings, with Google Scholar having the highest retrieval rate.

**Conclusion:**

This scientometric analysis highlights the research contributions and growth trajectory of clinical pharmacy and practice faculty, emphasising quality and opportunities for future advancements. The findings provide a benchmark for regional and global trends in clinical pharmacy research and offer actionable insights for researchers, academic institutions, and policymakers.

## Background

The primary aim of research is to generate new knowledge by transforming intangible inputs into tangible outputs like publications (Pal & Sarkar, 2020). As global research output expands, researchers and academic institutions face the challenge of evaluating the impact of their work on scientific and practical advancements (Cruz Rivera et al., 2017; Faraoni et al. 2023). Scientometric analysis has become a valuable tool for assessing research productivity, synthesising findings, and evaluating contributions of researchers and institutions (Abramo & D’Angelo, 2014; Castanha & Grácio, 2014; López-Pernas et al., 2022; Mejia et al., 2021; Sillet, 2013). It provides a comprehensive understanding of research output, identifies key knowledge indicators, and facilitates the identification of gaps and opportunities, paving the way for new insights (Abramo & D’Angelo, 2014; Castanha & Grácio, 2014; López-Pernas et al., 2022; Mejia et al., 2021; Sillet, 2013).

In the context of higher education, understanding and evaluating faculty research productivity is crucial for assessing institutional vision, research priorities, and individual contributions. This evaluation supports decisions regarding academic status, recognition, employment, promotions, tenure, and funding (Dorgu & James, 2019; Ryazanova & Jaskiene, 2022). Consequently, scientometric analysis provides a cost-effective, objective, and informative approach for analysing research output and assessing its impact through various indicators (López-Pernas et al., 2022; John Mingers & Leydesdorff, 2015). At the institutional level, it offers valuable insights into publication trends, areas of interest, and facilitates benchmarking among academic institutions (Abramo & D’Angelo, 2011; Gomis et al., 2023; John Mingers & Leydesdorff, 2015). This translates into practice as academia drives change through promoting research culture and mentoring junior researchers (Awaisu & Alsalimy, 2015; Sandgren, 2021).

In Qatar, research plays a prominent role, with Qatar University (QU) being a key contributor (Ministry of Education and Higher Education - State of Qatar, 2020). QU, the sole fully public-funded university in Qatar with over 1,000 faculty members, ranks second among Arab universities and within the top 101–150 globally in pharmacy/pharmacology according to the *Times Higher Education (THE)* and the Quacquarelli Symonds (QS) rankings (Quacquarelli Symonds (QS), 2021; Times Higher Education, 2023a, 2023b; TopUniversities, 2025). The College of Pharmacy at QU offers the only undergraduate and postgraduate pharmacy programmes in Qatar, and is equipped with state-of-the-art facilities, an ever-expanding research infrastructure, and the first international pharmacy programme to receive accreditation from the Canadian Council for Accreditation of Pharmacy Programs (CCAPP) in 2011 (College of Pharmacy - Qatar University, 2024). The expanding pharmacy field and evolving role of pharmacists have increased the commitment to publish research, advancing the profession and improving therapeutic outcomes (Alsharif, 2017; Dolovich & Tsuyuki, 2016; Sweileh, 2021). This trend has driven the development of clinical pharmacy and practice discipline as a vital research discipline focused on enhancing patients’ health outcomes (Jacobi, 2016; Jebara et al., 2021).

Although several bibliometric studies have analysed research output at regional and national levels (Abiib et al., 2021; Aguiar et al., 2021; Alotaibi et al., 2022; Idoudi et al., 2021; Sweileh, 2021; Sweileh et al., 2014), including some in Qatar, most have focused on general pharmacy research or broader disciplines (Sandgren, 2021; Y. Wang et al., 2022). This leaves gaps in understanding the nuanced and multifaceted clinical pharmacy and practice discipline. A comprehensive analysis of research output from a clinical pharmacy department is needed to capture its evolution, reflect on current trends, and identify key areas for future research (Gomis et al., 2023). This need is particularly evident given the advancements in the clinical pharmacy and practice field and the Qatar's evolving research infrastructure.

This study aimed to investigate the research productivity and quality of a clinical pharmacy and practice department at a Middle Eastern university using scientometric methods. Thus, this study would provide insights into the current state of departmental research, highlighting its contribution to improving health outcomes and advancing the global clinical pharmacy field.

## Methods

### Data sources

**Figure 1.**
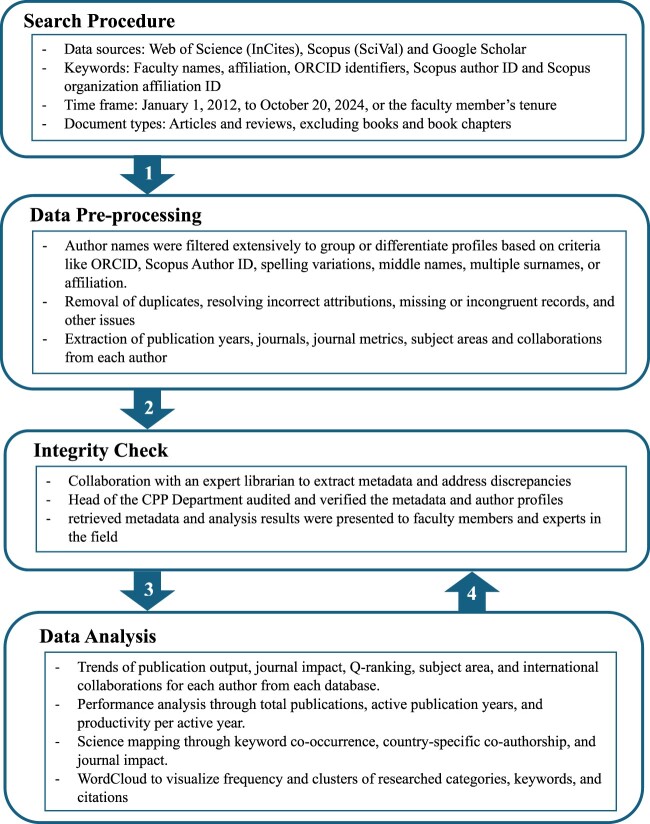
Scientometric data collection and analysis flowchart.

A systematic search was performed for all faculty members in the Department of Clinical Pharmacy and Practice (CPP) using three major scientometric databases: Web of Science (WoS) via InCites, Scopus via SciVal, and Google Scholar (Figure 1). WoS, a comprehensive subscription-based platform by Clarivate, and its analytics tool, InCites, offer reliable indicators for benchmarking global research output and performance across various disciplines (Birkle et al., 2020; Matthews, 2021). Scopus, a multidisciplinary, peer-reviewed abstract and citation database by Elsevier, and its analytics counterpart, SciVal, provide web-based analytics on institutional and researcher performance using comprehensive and flexible metrics (Wilsdon, 2016). Google Scholar was included for its broader coverage of research from non-indexed journals, bilingual publications, and other alternative sources especially in the Global South (Aguillo, 2012; Pereira & Mugnaini, 2023). Lacking its own journal impact assessment, the SCImago Journal Rank (SJR2) indicator was used. SCImago, an open-access platform, covers over 5,000 global publishers and is maintained by a Spanish research group collaborating with institutions such as Consejo Superior de Investigaciones Científicas (CSIC), Universidad de Granada, and several universities (Fernandez-Llimos, 2018; John Mingers & Leydesdorff, 2015; Vicente, 2012). Utilising multiple databases overcomes the limitations of any single source, providing a robust and reliable assessment of research productivity within the field of clinical pharmacy and practice (Blakeman, 2018; Castanha & Grácio, 2014; García-Villar & García-Santos, 2021; López-Pernas et al., 2022; Pereira & Mugnaini, 2023).

### Scientometric indicators

Quantitative and qualitative scientometric indicators were employed per established best practices, to provide a comprehensive analysis of faculty research output (Abramo & D’Angelo, 2011; López-Pernas et al., 2022; John Mingers & Leydesdorff, 2015; Oosthuizen & Fenton, 2014). This section briefly describes the indicators used in the scientometric analysis, including publication output, journal impact metrics, Q-ranking, subject area analyses, and collaboration patterns.

1. Yearly research output: Total and annual publication data were obtained for each faculty member’s tenure or the study period (2012–2024), whichever occurred first.
2. Journal impact assessment: For WoS, impact factor (IF) was used, which is calculated as the ratio of citations a journal receives in a given year for articles published in the previous two years to the total number of citable documents indexed in WoS during the same period (Fernandez-Llimos, 2018). For Scopus, CiteScore was employed, which calculates the ratio of citations a journal receives in a year for documents published during the previous four years to the total number of documents indexed in Scopus during the same period (Fernandez-Llimos, 2018). For Google Scholar, the SJR2 indicator was used which employs a field-weighted citation impact method based on the Google PageRank algorithm (Vicente, 2012). It measures a publication's citation count compared to similar publications within the same subject area, document type, and year (Fernandez-Llimos, 2018; López-Pernas et al., 2022). A field-weighted citation impact score of 1.0 represents the global average, with scores above 1.0 indicating higher citation frequency and scores below 1.0 indicating lower citation frequency (Vicente, 2012).
3. Q-ranking: Another common scientometric indicator is a journal’s Q-rank, which categorises journals into quartiles (Q1 to Q4) in a specific field based on their relative impact, citations, and indexing (López-Pernas et al., 2022). Q-ranking values may vary across databases due to differences in journal impact metrics, indexed content, year ranges, and citation data (Wilsdon, 2016).
4. Subject area analysis: keyword, category, and citation topic co-occurrence data from each database were analysed to provides insights into the research focus areas trending or emerging topics, recurring themes, and concepts (You et al., 2024).
5. Collaborations: National, regional, and international collaborations were analysed to identify primary collaborating countries and quantify joint global research efforts, providing insights into the global research network in clinical pharmacy and practice. Furthermore, mapping the geographical distribution of research collaborations can help to identify areas where collaboration is robust and those requiring further developments to advance the field more comprehensively.

### Search procedure

#### Data retrieval

Data were collected to assess the CPP faculty research productivity in collaboration with a QU librarian. The scientometric analysis covered the period from 1 January 2012, to 20 October 2024, or the faculty member’s tenure at QU, whichever occurred first. The 12-year timeframe was selected based on literature recommendations for a citation window of at least five years for accurate evaluations (Nederhof et al., 2012; Wang, 2012). An Author/Researcher search was conducted using faculty name, affiliation, ORCID identifier, Scopus author ID, and Scopus organization affiliation ID (QU ID 25990 and College of Pharmacy ID 60197118). Prioritising ORCID, Scopus author ID, and affiliation IDs minimised the risk of incorporating unrelated researchers due to possible name spelling variations or similarities.

Metadata were retrieved by two authors (IZ and MT) and reviewed by a third author (AA), who was also consulted in instances of disagreement or discrepancy. Documents indexed in WoS, Scopus, and Google Scholar were included, excluding books and book chapters. InCites (WoS) provided publication counts, journal impact factors, Q-rankings, and subject area categories. SciVal (Scopus) provided publication counts, CiteScore, Q-rankings, international collaboration data, and Topic WordClouds. Similarly, Google Scholar provided publications and subject areas. Due to the absence of a built-in metadata scraping feature in Google Scholar, metadata was manually and meticulously extracted from each verified Google Scholar profile for the period spanning 2012–2024. This data was subsequently transferred into a CSV structured format for manual input into Excel, as described in the literature (Costa et al., 2024; Suominen et al., 2018). SCImago Journal and Country Ranking offered SJR2 journal impact and Q-ranking metrics from Google Scholar metadata.

#### Data pre-processing

All extracted data were entered into Microsoft Excel for revision, filtering, and debugging by the authors. Data pre-processing was manually conducted to verify, refine, and remove duplicates, resolve incorrect attributions, missing or incongruent records, minimiz the noise in the retrieved records, and ensure data eligibility for analysis (López-Pernas et al., 2022). Author names were filtered extensively to group or differentiate profiles based on criteria like ORCID, Scopus Author ID, spelling variations, middle names, multiple surnames, or affiliation. These steps ensured data accuracy and reliability, enabling robust scientometric analysis.

### Data analysis

A descriptive analysis summarised research productivity, including publication output, journal impact, Q-ranking, subject area analysis, and international collaborations for each database. Performance analysis techniques included total publications, active publication years, and productivity per active year. Science mapping techniques included keyword co-occurrence, country-specific co-authorship, and journal impact analyses. Citation-based metrics are presented using percentiles, medians, and interquartile ranges (IQR) due to skewed citation distributions, in line with established scientometric practices (Bornmann & Mutz, 2011; Leydesdorff & Milojevic, 2012; Opthof & Leydesdorff, 2010; van Raan et al., 2010). Results are presented graphically and visualised in WordCloud to identify clusters of frequently researched categories, keywords, and citations within the field of clinical pharmacy and practice. WordCloud facilitates the visualisation of the top 50 subject area keywords, with centrality, size, and color representing the relative frequency of each term compared to others to identify thematic areas, research patterns, and emerging trends in the field.

### Integrity check

The integrity of the retrieved metadata was verified through collaboration with a librarian, ensuring data accuracy and consistency across different databases. The Head of the CPP Department audited and verified the metadata and author profiles, addressing any discrepancies as appropriate. Finally, the retrieved metadata and analysis results were presented to faculty members and experts in the field to assess the alignment with the current state of research in the field.

## Results

### Web of Science (WoS)

**Figure 2.**
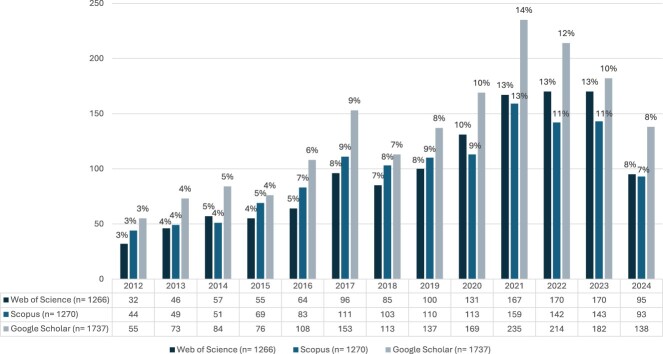
Year-wise distribution of articles per database between 2012 and 2024.

During the 12-year period, 31 CPP faculty members published 1,266 articles in 293 sources (Figure 2). Faculty tenure ranged from 2 to 18 years, with an average of 10 years. On average, faculty members published 41 articles during their tenure at Qatar University, with an annual mean publication rate of 5 articles (Table 1). Faculty members had active publication years for 85% ± 23% of their tenure. The average annual growth rate (AAGR) for publications was 13%, with the highest publication rates between 2021 and 2023, averaging over 160 publications annually.

**Table 1.** Clinical Pharmacy and Practice Department faculty publication distribution per year.

Faculty	Total duration (years)	Total publications while active at QU			Average publications per year per tenure		
		WoS	Scopus	Scholar	WoS	Scopus	Scholar
1	13	168	179	257	13	14	20
2	13	39	49	74	3	4	6
3	6	48	30	73	8	5	12
4	15	22	24	42	1	2	3
5	13	23	26	28	2	2	2
6	8	15	18	23	2	2	3
7	14	73	68		5	5	0
8	6	148	104	207	25	17	35
9	3	12	8	16	4	3	5
10	3	9	9	13	3	3	4
11	9	5	6	7	1	1	1
12	12	72	60	90	6	5	8
13	6	12	13	13	2	2	2
14	10	42	42	51	4	4	5
15	7	65	89	104	9	13	15
16	17	67	54	68	4	3	4
17	11	10	20		1	2	0
18	13	218	226	308	17	17	24
19	11	40	48	74	4	4	7
20	4	27	26	38	7	7	10
21	5	1	4	2	0	1	0
22	7	20	18	31	3	3	4
23	10	28	32	56	3	3	6
24	29	3	3	4	0	0	0
25	7	11	19	28	2	3	4
26	9	14	17	20	2	2	2
27	3	6	7	9	2	2	3
28	2	14	13	14	7	7	7
29	11	16	15	21	1	1	2
30	7	21	22	43	3	3	6
31	11	17	21	23	2	2	2

#### Journal impact (IF) and Q-ranking

CPP faculty members published in 293 different journals, with the top 10 targeted journals being tabulated in Table 2. *International Journal of Clinical Pharmacy* was the most frequently cited (over 150 publications), followed by *Research in Social and Administrative Pharmacy* and *PLOS One* (Table 2). The top 10 journals reflect the broad and multidisciplinary nature of the CPP department’s research activities, as these journals are categorised under the WoS categories of ‘*Pharmacy/Pharmacology*’, ‘*Public Environmental Occupational Health*’, ‘*Multidisciplinary Sciences*’, ‘*Education Scientific Disciplines*’, ‘*Economics*’, ‘*Health Care Sciences Services*’ and ‘*Health Policy Services*’. The majority of these journals are published by American or European entities, with the exception of the *Saudi Pharmaceutical Journal*.

**Table 2.** The most widely targeted publication sources in each database.

Journal		Publications	Impact factor	Q-ranking
**Web of Science**				
1	International Journal of Clinical Pharmacy	158	2.6	2
2	Research in Social and Administrative Pharmacy	62	1.7	2
3	PLOS One	45	3.8	2
4	American Journal of Pharmaceutical Education	38	3.8	1
5	BMC Medical Education	29	3.4	1
6	Value in Health	27	6.2	1
7	Saudi Pharmaceutical Journal	26	3.9	1
8	Current Problems in Cardiology	23	4.3	2
9	Journal of Pharmaceutical Policy and Practice	22	3.7	1
10	Pharmacotherapy	22	4	2
	Journal	Publications	CiteScore	Q-ranking
**Scopus**				
1	International Journal of Clinical Pharmacy	101	4.1	1
2	Research in Social and Administrative Pharmacy	59	7.2	1
3	American Journal of Pharmaceutical Education	39	4.3	1
4	Currents in Pharmacy Teaching and Learning	38	2.1	2
5	PLOS ONE	37	6.2	1
6	BMC Medical Education	30	4.9	1
7	Journal of Pharmaceutical Health Services Research	27	1.5	2
8	Pharmacy Education	26	0.8	3
9	Saudi Pharmaceutical Journal	23	6.1	2
10	Current Problems in Cardiology	22	4.8	2
	Journal	Publications	SJR2	Q-ranking
**Google Scholar**				
1	International Journal of Clinical Pharmacy	168	0.577	1
2	Research in Social and Administrative Pharmacy	74	0.833	1
3	Value in Health	46	1.507	1
4	American Journal of Pharmaceutical Education	41	1.11	1
5	PLOS One	41	0.839	1
6	Currents in Pharmacy Teaching and Learning	36	0.45	2
7	BMC Medical Education	32	0.935	1
8	Saudi Pharmaceutical Journal	26	0.589	2
9	Journal of Pharmaceutical Health Services Research	23	0.206	3
10	International Journal of Pharmacy Practice	22	0.493	1

**Figure 3.**
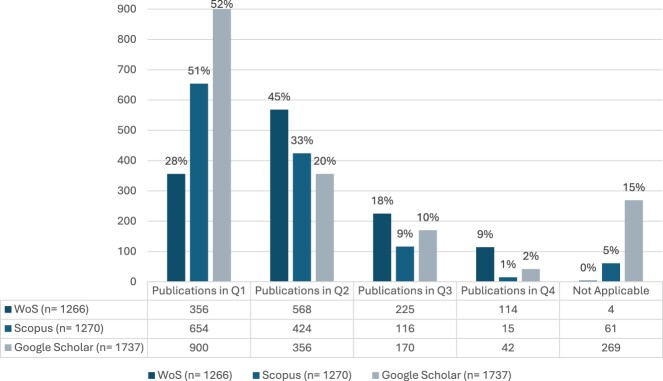
Publications of CCP Department based on Q-ranking (2012–2024).

Journal IF showed that 48% of publications were in journals with an IF of 1.00–2.99, 37% in journals with an IF of 3.00–4.99, and 10% in journals with an IF ≥5.00 (Table 3). It is noteworthy that quantity does not always correlate with quality. For instance, one faculty member, contributing 5% of the total publication output (n =  65), had over 20% of their publications in journals with an impact factor above 5.00. Regarding Q-ranking, the majority of the publications were in Q2 journals (45%), followed by Q1 (28%), and Q3 (18%) (Figure 3). The median percentage of each faculty member’s publications in Q1 journals was 30% (IQR = 16%), with nearly 30% of the department’s total publications in Q1 journals over the 12-year period.

**Table 3.** CPP publications by journal impact between WoS and Scopus (2012–2024).

Faculty	<1		1.00–2.99		3.00–4.99		≥ 5.00		Not applicable	
	IF	CiteScore	IF	CiteScore	IF	CiteScore	IF	CiteScore	IF	CiteScore
1	4 (7%)	10 (21%)	79 (13%)	24 (11%)	65 (14%)	67 (15%)	20 (16%)	58 (13%)	0 (0%)	20 (21%)
2	2 (3%)	1 (2%)	22 (4%)	6 (3%)	15 (3%)	21 (5%)	0 (0%)	18 (4%)	0 (0%)	3 (3%)
3	0 (0%)	1 (2%)	29 (5%)	9 (4%)	13 (3%)	7 (2%)	5 (4%)	13 (3%)	1 (4%)	0 (0%)
4	2 (3%)	3 (6%)	9 (1%)	1 (0%)	11 (2%)	9 (2%)	0 (0%)	6 (1%)	0 (0%)	5 (5%)
5	0 (0%)	0 (0%)	15 (2%)	6 (3%)	7 (1%)	9 (2%)	1 (1%)	9 (2%)	0 (0%)	2 (2%)
6	0 (0%)	1 (2%)	7 (1%)	6 (3%)	6 (1%)	3 (1%)	2 (2%)	8 (2%)	0 (0%)	0 (0%)
7	0 (0%)	0 (0%)	24 (4%)	7 (3%)	40 (8%)	30 (7%)	9 (7%)	28 (6%)	0 (0%)	3 (3%)
8	6 (10%)	4 (8%)	79 (13%)	6 (3%)	38 (8%)	42 (9%)	25 (20%)	49 (11%)	0 (0%)	3 (3%)
9	0 (0%)	1 (2%)	8 (1%)	1 (0%)	4 (1%)	4 (1%)	0 (0%)	2 (0%)	0 (0%)	0 (0%)
10	2 (3%)	0 (0%)	4 (1%)	3 (1%)	3 (1%)	6 (1%)	0 (0%)	0 (0%)	0 (0%)	0 (0%)
11	0 (0%)	0 (0%)	3 (0%)	0 (0%)	2 (0%)	6 (1%)	0 (0%)	0 (0%)	0 (0%)	0 (0%)
12	0 (0%)	0 (0%)	34 (6%)	6 (3%)	34 (7%)	24 (5%)	4 (3%)	30 (7%)	0 (0%)	0 (0%)
13	1 (2%)	1 (2%)	4 (1%)	2 (1%)	7 (1%)	4 (1%)	0 (0%)	6 (1%)	0 (0%)	0 (0%)
14	0 (0%)	2 (4%)	13 (2%)	5 (2%)	15 (3%)	19 (4%)	12 (10%)	15 (3%)	2 (8%)	1 (1%)
15	1 (2%)	4 (8%)	30 (5%)	22 (10%)	18 (4%)	24 (5%)	14 (11%)	35 (8%)	2 (8%)	4 (4%)
16	2 (3%)	1 (2%)	45 (7%)	5 (2%)	16 (3%)	27 (6%)	4 (3%)	18 (4%)	0 (0%)	3 (3%)
17	0 (0%)	1 (2%)	6 (1%)	1 (0%)	3 (1%)	8 (2%)	1 (1%)	9 (2%)	0 (0%)	1 (1%)
18	37 (63%)	10 (21%)	87 (14%)	66 (31%)	85 (18%)	63 (14%)	8 (6%)	56 (12%)	0 (0%)	31 (33%)
19	0 (0%)	1 (2%)	15 (2%)	15 (7%)	25 (5%)	9 (2%)	0 (0%)	23 (5%)	0 (0%)	0 (0%)
20	0 (0%)	0 (0%)	11 (2%)	1 (0%)	13 (3%)	8 (2%)	3 (2%)	17 (4%)	0 (0%)	0 (0%)
21	0 (0%)	0 (0%)	0 (0%)	0 (0%)	1 (0%)	0 (0%)	0 (0%)	1 (0%)	0 (0%)	3 (3%)
22	0 (0%)	0 (0%)	11 (2%)	1 (0%)	8 (2%)	6 (1%)	1 (1%)	10 (2%)	20 (80%)	1 (1%)
23	0 (0%)	1 (2%)	19 (3%)	5 (2%)	6 (1%)	15 (3%)	3 (2%)	8 (2%)	0 (0%)	3 (3%)
24	0 (0%)	1 (2%)	1 (0%)	0 (0%)	0 (0%)	1 (0%)	2 (2%)	1 (0%)	0 (0%)	0 (0%)
25	0 (0%)	3 (6%)	4 (1%)	4 (2%)	4 (1%)	6 (1%)	3 (2%)	4 (1%)	0 (0%)	2 (2%)
26	0 (0%)	0 (0%)	6 (1%)	0 (0%)	6 (1%)	6 (1%)	2 (2%)	6 (1%)	0 (0%)	5 (5%)
27	0 (0%)	0 (0%)	5 (1%)	4 (2%)	1 (0%)	3 (1%)	0 (0%)	0 (0%)	0 (0%)	0 (0%)
28	1 (2%)	0 (0%)	5 (1%)	0 (0%)	7 (1%)	5 (1%)	1 (1%)	8 (2%)	0 (0%)	0 (0%)
29	0 (0%)	0 (0%)	9 (1%)	3 (1%)	4 (1%)	3 (1%)	3 (2%)	5 (1%)	0 (0%)	4 (4%)
30	0 (0%)	1 (2%)	13 (2%)	2 (1%)	7 (1%)	6 (1%)	1 (1%)	13 (3%)	0 (0%)	0 (0%)
31	1 (2%)	1 (2%)	8 (1%)	5 (2%)	8 (2%)	10 (2%)	0 (0%)	5 (1%)	0 (0%)	0 (0%)
Total	59 (5%)	48 (4%)	605 (48%)	216 (17%)	472 (37%)	451 (36%)	124 (10%)	461 (36%)	25 (2%)	94 (7%)

#### Subject area analysis (WoS categories)

**Figure 4.**
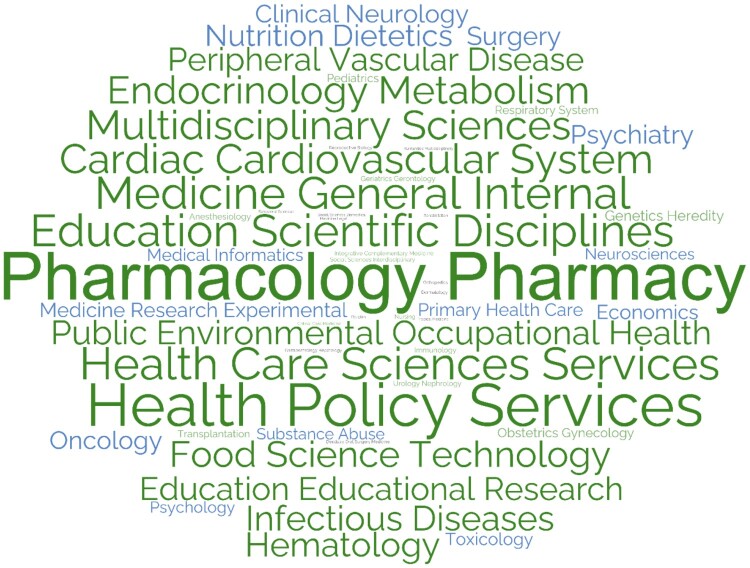
Top 50 categories word cloud of CCP Department based on WoS categories (2012–2024).

Faculty addressed over 130 categories, with more than 3,000 co-occurrences. The most prevalent categories by frequency were explored and presented in the Categories WordCloud (Figure 4). The most frequent categories included ‘*Pharmacology/Pharmacy*’ with 819 records (27%), ‘*Health Care Sciences Services*’ with 171 records (6%), ‘*Public Environmental Occupational Health*’ with 166 records (5%), ‘*Education Scientific Disciplines*’ with 158 records (5%) and ‘*Health Policy Services*’ with 134 records (4%). The faculty published 51% of their research output in *Life Sciences & Biomedicine*, 24% in *Social Sciences*, 23% in *Technology*, and 2% in *Arts and Humanities* research Core Categories.

#### Collaborations

**Figure 5.**
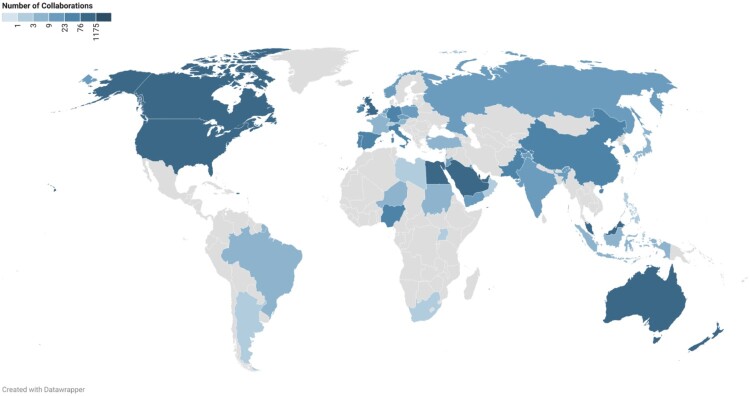
Geographical distribution of CCP Department collaborations based on WoS (2012–2024).

Collaborations were highest within the local community (31%), followed by Scotland (10%) and Egypt (9%) (Figure 5). Approximately 52% of the department’s collaborations were international, while 17% were regional. Academic-Corporation collaborations comprised 1.2% of the department's total research output.

### Scopus

Over the 12-year period, 31 faculty members produced 1,270 articles indexed in Scopus (Figure 1), averaging five publications per year and 41 publications per faculty member during their tenure at QU (Table 1). Faculty members maintained active publication status for 86% ± 20% of their tenure duration. Research output increased steadily with an AAGR of 14%. The highest publications output was recorded in 2021, with nearly 160 articles published by the department members.

#### Journal impact (CiteScore) and Q-ranking

Publications were disseminated in 281 journals, with the *International Journal of Clinical Pharmacy* being the most targeted journal (over 100 publications), followed by *Research in Social and Administrative Pharmacy* and the *American Journal of Pharmaceutical Education* journals (Table 2). The top 10 targeted journals frequently cover core topics in clinical pharmacy and practice, categorised under: ‘*Pharmacy*’, ‘*Education*’, ‘*Multidisciplinary*’, ‘*General Medicine*’ and ‘*Economics, Econometrics and Finance*’, among others. Similar to WoS, the majority of the top targeted journals were published by American or European entities, except for *Saudi Pharmaceutical Journal*. CiteScore showed that 36% of publications were in journals with a CiteScore of ≥5.00, 36% between 3.00 and 4.99, and 17% between 1.00 and 2.99 (Table 3). This trend was consistent with the individual faculty output, with over 30% of their publications in journals with a CiteScore of ≥5.00 (IQR = 21%). In terms of Q-ranking, 51% were in Q1 journals, 33% in Q2 journals and 9% in Q3 journals (Figure 3). Individual faculty publications mirrored this pattern, with over 50% of their individual output in Q1 journals (IQR = 23%) (Table 4).

**Table 4.** CPP publications in Q1 journals as per WoS, Scopus and SCImago Journal and Country Rank for Google Scholar metadata (2012–2024).

Faculty	Publications in Q1		
	WoS	Scopus	Google Scholar
AAW	47 (13%)	91 (14%)	147 (16%)
AEA	12 (3%)	38 (6%)	38 (4%)
AAH	24 (7%)	12 (2%)	38 (4%)
BAM	6 (2%)	13 (2%)	15 (2%)
BPJ	7 (2%)	15 (2%)	15 (2%)
DCR	4 (1%)	11 (2%)	12 (1%)
DAB	21 (6%)	28 (4%)	0 (0%)
DCS	45 (13%)	84 (13%)	144 (16%)
EKB	3 (1%)	2 (0%)	8 (1%)
FAK	3 (1%)	3 (0%)	4 (0%)
HAE	2 (1%)	2 (0%)	3 (0%)
HFE	5 (1%)	24 (4%)	33 (4%)
KBY	3 (1%)	7 (1%)	7 (1%)
KLW	23 (6%)	24 (4%)	29 (3%)
KJW	27 (8%)	41 (6%)	39 (4%)
MSE	12 (3%)	38 (6%)	47 (5%)
MID	4 (1%)	13 (2%)	0 (0%)
MIM	46 (13%)	75 (11%)	110 (12%)
MZL	10 (3%)	18 (3%)	20 (2%)
MAH	11 (3%)	21 (3%)	29 (3%)
MAI	1 (0%)	1 (0%)	2 (0%)
MEJ	3 (1%)	14 (2%)	26 (3%)
ANK	5 (1%)	20 (3%)	34 (4%)
RAM	2 (1%)	1 (0%)	2 (0%)
SPK	6 (2%)	10 (2%)	16 (2%)
SSK	6 (2%)	7 (1%)	12 (1%)
TAD	1 (0%)	3 (0%)	4 (0%)
UAB	3 (1%)	8 (1%)	6 (1%)
YBO	4 (1%)	2 (0%)	13 (1%)
ZJN	3 (1%)	18 (3%)	36 (4%)
ZNS	7 (2%)	10 (2%)	11 (1%)
Total	356 (28%)	654 (51%)	900 (52%)

#### Subject area keywords (Scopus topics)

**Figure 6.**
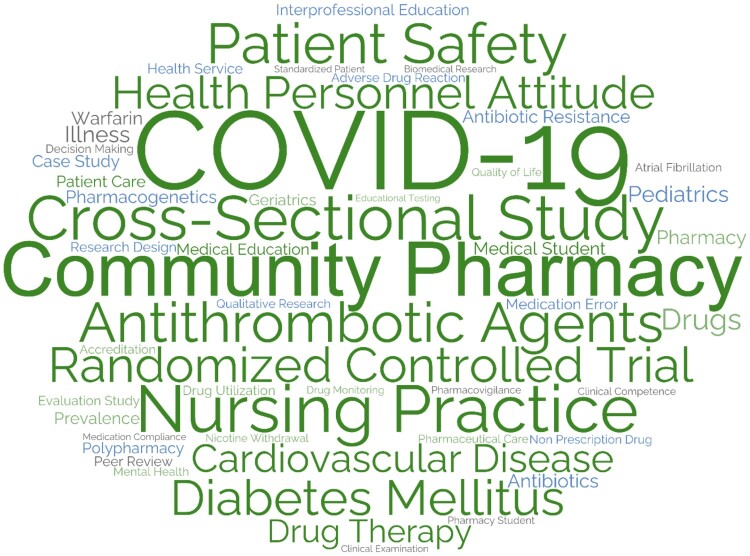
Citation topic WordCloud of the top 50 topics of CCP Department based on Scopus Topics (2012–2024).

The faculty addressed over 450 topics with almost 2,500 co-occurrences. The most prevalent topics by frequency on SciVal were examined and visualised in the Topic WordCloud (Figure 6). Key topics included ‘*Community Pharmacy*’ with 134 documents (5.5%), ‘*COVID-19*’ with 109 documents (4.5%), ‘*Drug Therapy*’ with 59 documents (2.4%), ‘*Patient Safety*’ with 58 documents (2.4%), and ‘*Diabetes Mellitus*’ with 46 documents (1.9%). SciVal also highlighted several topics where faculty articles were cited above the global average, including ‘*COVID-19*’ (174 documents), ‘*Research Design*’ (36 documents), ‘*Meta-Analysis*’ (30 documents), ‘*Community Pharmacy*’ (21 documents), and ‘*Anti-infective Agents*’ (12 documents).

#### Collaborations

The analysis of these collaborations showed that 53% of the department’s publications involved international collaborations, while about 40% involved national collaborations. Academic-Corporate collaborations averaged 1.8% of total research output.

### Google scholar

A total of 1,737 publications were retrieved from Google Scholar across 29 faculty members, as two faculty members did not have profiles (Figure 2). The average annual output was seven publications, totaling 60 publications per faculty member (Table 1). Faculty were active in publishing for 83% ± 30% of their tenure. The AAGR increased by 12% over 12 years, with 2021 being the peak year, producing over 200 publications.

#### Journal impact (SJR2) and Q-ranking

Google Scholar recorded 356 journals where faculty published their research, the top 10 of those are in Table 2. Again, the *International Journal of Clinical Pharmacy* was the most targeted journal, with over 160 publications, followed by *Research in Social and Administrative Pharmacy* and *Value in Health* journals (Table 2). The top 10 journals primarily covered subject areas such as ‘*Health Policy*,’ ‘*Public Health, Environmental and Occupational Health*,’ ‘*Medicine*,’ ‘*Education*,’ and ‘*Pharmacy*.’ The Saudi Pharmaceutical Journal was the only top-targeted journal from a global south entity. Using the SJR2 field-weighted citation impact indicator, 35% of the research was published in journals with citation counts 20–60% below the global average, 30% in journals with average global citations, and 10% in journals above the global average (Table 5). An average of 20% of individual faculty publications were in journals with higher-than-average global citations. Google Scholar also revealed a higher number of journals not indexed in the SCImago Journal and Country Rank database, with nearly 15% of departmental publications in un-indexed journals. However, most publications were in Q1 journals (52%), followed by Q2 (20%) and 10% in Q3 (Figure 3).

**Table 5.** CPP Publications by journal impact as per SCImago Journal and Country Rank based on Google Scholar metadata (2012–2024).

Faculty	Total while active at QU	Journal with SJR2 <0.399	Journal with SJR2 0.400–0.799	Journal with SJR2 0.800–1.199	Journal with SJR2 ≥ 1.200	Not applicable
1	257 (15%)	26 (12%)	79 (13%)	71 (15%)	33 (21%)	48 (18%)
2	74 (4%)	6 (3%)	13 (2%)	32 (7%)	0 (0%)	23 (9%)
3	73 (4%)	1 (0%)	19 (3%)	17 (4%)	23 (14%)	13 (5%)
4	42 (2%)	9 (4%)	11 (2%)	8 (2%)	1 (1%)	13 (5%)
5	28 (2%)	1 (0%)	15 (2%)	8 (2%)	2 (1%)	2 (1%)
6	23 (1%)	4 (2%)	8 (1%)	7 (1%)	3 (2%)	1 (0%)
7	207 (12%)	18 (8%)	80 (13%)	70 (15%)	12 (8%)	27 (10%)
8	16 (1%)	2 (1%)	10 (2%)	2 (0%)	0 (0%)	2 (1%)
9	13 (1%)	3 (1%)	3 (0%)	4 (1%)	0 (0%)	3 (1%)
10	7 (0%)	1 (0%)	3 (0%)	3 (1%)	0 (0%)	0 (0%)
11	90 (5%)	3 (1%)	45 (7%)	26 (5%)	5 (3%)	11 (4%)
12	13 (1%)	2 (1%)	6 (1%)	4 (1%)	1 (1%)	0 (0%)
13	51 (3%)	6 (3%)	19 (3%)	16 (3%)	7 (4%)	3 (1%)
14	104 (6%)	16 (8%)	42 (7%)	25 (5%)	6 (4%)	15 (6%)
15	68 (4%)	1 (0%)	31 (5%)	28 (6%)	4 (3%)	4 (1%)
16	308 (18%)	77 (36%)	88 (14%)	62 (13%)	18 (11%)	63 (23%)
17	74 (4%)	16 (8%)	22 (4%)	20 (4%)	7 (4%)	9 (3%)
18	38 (2%)	0 (0%)	19 (3%)	13 (3%)	3 (2%)	3 (1%)
19	2 (0%)	0 (0%)	0 (0%)	0 (0%)	2 (1%)	0 (0%)
20	31 (2%)	2 (1%)	9 (1%)	18 (4%)	1 (1%)	1 (0%)
21	56 (3%)	8 (4%)	21 (3%)	8 (2%)	10 (6%)	9 (3%)
22	4 (0%)	0 (0%)	1 (0%)	0 (0%)	2 (1%)	1 (0%)
23	28 (2%)	3 (1%)	11 (2%)	7 (1%)	2 (1%)	5 (2%)
24	20 (1%)	0 (0%)	9 (1%)	3 (1%)	6 (4%)	2 (1%)
25	9 (1%)	2 (1%)	7 (1%)	0 (0%)	0 (0%)	0 (0%)
26	14 (1%)	2 (1%)	7 (1%)	3 (1%)	2 (1%)	0 (0%)
27	21 (1%)	2 (1%)	9 (1%)	3 (1%)	6 (4%)	1 (0%)
28	43 (2%)	0 (0%)	16 (3%)	17 (4%)	3 (2%)	7 (3%)
29	23 (1%)	2 (1%)	11 (2%)	6 (1%)	1 (1%)	3 (1%)
Total	1737 (100%)	213 (12%)	614 (35%)	481 (28%)	160 (9%)	269 (15%)

#### Subject area keywords (Google Scholar profile keywords)

**Figure 7.**
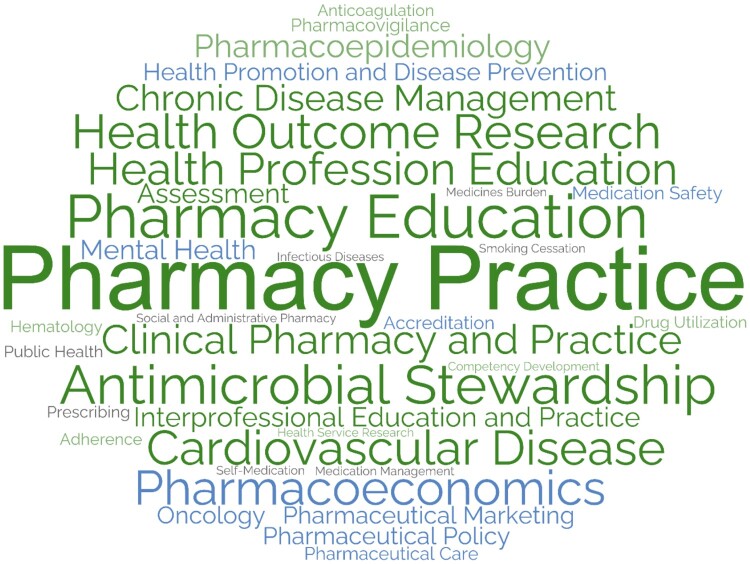
Keywords WordCloud of the Top 35 topics of CCP Department faculty based on Google Scholar profile (2012–2024).

More than 50 keywords were linked to faculty members profiles, with about 80 co-occurrences. The 35 most prevalent and unique keywords were examined and are presented in the keyword WordCloud (Figure 7). The most frequently occurring keywords included ‘*Pharmacy Practice*’ for 8 faculty members (28%), ‘*Pharmacy Education*’ for 6 faculty members (21%), ‘*Antimicrobial Stewardship*’ for 4 faculty members (14%), ‘*Cardiovascular Disease*’ for 3 faculty members (10%) and ‘*Clinical Pharmacy*’ for 3 faculty members (10%).

### Comparison between the databases (WoS, Scopus, and Google Scholar)

It was crucial to compare the results from the three databases by highlighting the similarities and differences in scientometric indicators of publications, journal impact, Q-ranking of indexed journals, and subject areas.

#### Publications

The total number of publications exhibited minimal variation between WoS (*n* = 1,266) and Scopus (*n* = 1,270), while Google Scholar had 37% more publications (*n* = 1,737). Table 6 presents the top 10 publishing faculty members across the databases. WoS captured more faculty publications from 2019 to 2024, averaging 12% higher annually than Scopus, whereas Scopus had 23% more publications annually in earlier years. An anomalous case in WoS due to one researcher whose author profile erroneously combined publications from other researchers with similar names, which necessitated the manual removal of 136 falsely attributed publications. This error was not present in Scopus or Google Scholar Faculty with longer affiliations had more publications in Scopus than in WoS. Over 70% of faculty with 5–9 and 10–14 years of affiliation had more publications in Scopus, compared to 25% of those with 1–4 years.

**Table 6.** The top 10 publishing faculty in each database.

Faculty		Total WoS Publications	Faculty	Total Scopus Publications	Faculty	Total Google Scholar Publications
1	MIM	218	MIM	226	MIM	308
2	AAW	168	AAW	179	AAW	257
3	DCS	148	DCS	104	DCS	207
4	DAB	73	KJW	89	KJW	104
5	HFE	72	DAB	68	HFE	90
6	MSE	67	HFE	60	AEA	74
7	KJW	65	MSE	54	MZL	74
8	AAH	48	AEA	49	AAH	73
9	KLW	42	MZL	48	MSE	68
10	MZL	40	KLW	42	ANK	56

#### Journal impact

The *International Journal of Clinical Pharmacy* and *Research in Social and Administrative Pharmacy* were the most frequently targeted sources of publications across all databases. While the total number of publication sources varied among databases, 208 journals were shared among them. Of these, 102 journals (49%) had consistent publication metadata across databases, whereas 106 journals (51%) showed discrepancies. Google Scholar had the highest retrieval rate, with higher publications per faculty member for 26% of the shared journals. Scopus and WoS had similar retrieval rates for 20% of journals. Google Scholar consistently retrieved similar publication sources for Scopus (44%) and WoS (37%). Google Scholar retrieved the highest proportion of exclusive journals not indexed in either WoS or Scopus (70%), followed by WoS (18%), and Scopus (12%).

Direct comparison with SJR2 is challenging due to its nature as a weighted field citation impact method. Therefore, the comparison primarily focuses on the impact factor and CiteScore, which are more directly comparable. Scopus had a higher CiteScore in 88% of cases for the same journal compared to WoS, whereas WoS had a higher impact factor in only 3% of shared journals. The CiteScore and impact factor were identical in only three journals (1%). Over 80% of faculty members had more publications in journals with a CiteScore above 5.00 in Scopus than in WoS.

#### Q-ranking

The Q-ranking indicators can be comparatively analysed across the three databases. Among the metadata, 95 journals (44%) had identical Q rankings across databases. For the remaining 56% where ranking differed, Scopus and SCImago Journal and Country Ranking showed similar Q-ranking distribution in approximately 50% of cases. Scopus showed a higher Q-ranking for the same journals in about 20% of instances across the three databases. The faculty’s Q-ranking distributions indicated that SCImago had higher retrieval and representation rates for Q1 journals, while WoS excelled in retrieving Q2 – Q4 publications.

#### Subject area analysis

There was a substantial and anticipated overlap in subject area keywords across the three databases, predominantly related to pharmacy and its practice, health education, patient and medication safety, and chronic disease management (e.g. cardiovascular diseases, diabetes mellitus).

## Discussion

This scientometric analysis offers valuable insights into the research productivity and impact of the Department of Clinical Pharmacy and Practice at Qatar University from 2012 to 2024. The department's cumulative output, including 1,266 articles indexed in WoS, 1,270 in Scopus, and 1,737 in Google Scholar, underscores its growing scholarly contributions. The findings revealed a consistent upward increase in publication output, with a pronounced increase between 2021 and 2023, reflecting the department's commitment to advancing research aligned with institutional and national goals. Comparative analysis of databases revealed variations in retrieval rates and journal metrics, highlighting the importance of multi-database approaches in scientometric evaluations.

Google Scholar retrieved 37% more publications than other databases, consistent with its reported ability to capture 90% of research output across diverse subject areas (Adriaanse & Rensleigh, 2011; Martín-Martín et al., 2018). This advantage arises from several factors such as institutional scholarly output hosted on commercial or non-profit domains indexed by Google Scholar but often excluded by WoS and Scopus (Aguillo, 2012). Despite high global rankings in scientific output, non-English speaking countries remain disproportionately underrepresented in indexed journals, with nearly 80% of indexed journals published in English (Bahji et al., 2023; Gordin, 2015; Pakenham-Walsh, 2018; Pereira & Mugnaini, 2023; van Weijen Dr, 2012). This trend is especially prominent in Asian and Latin American universities, where Google Scholar indexes more publications than the other databases (Aguillo, 2012). This issue affects not only countries in the global south, but also underrepresented countries with established research infrastructure such as Australia, Canada, and some European countries despite their substantial growth in scientific productivity (Pereira & Mugnaini, 2023). Addressing this imbalance is crucial to fostering equitable global research representation.

While Google Scholar provides broader coverage, its data quality often requires manual verification due to inconsistencies. Prior studies report that Google Scholar metadata have 14% inconsistencies in author names, article titles, and dates, compared to 5.4% in WoS and 0.4% in Scopus (Adriaanse & Rensleigh, 2011; Harzing & Van der Wal, 2008; Jacso, 2005; John Mingers & Leydesdorff, 2015). Overall, the results of this scientometric analysis support the consensus that metadata from WoS Scopus are more reliable for evaluating research in peer-reviewed fields despite their limited retrieval and linguistic biases, whereas Google Scholar offers broader subject area and language coverage but exhibits challenges with retrieved data quality (John Mingers & Leydesdorff, 2015).

A notable trend is the correlation between longer tenure and preference for publishing in Scopus-indexed journals. Google Scholar’s broader coverage captures more publications, but complicates accurate quantification of research impact for institutional evaluations (J. Mingers et al., 2017). Scopus’ higher metadata accuracy likely explains its use by QU for evaluating faculty scientific output, influencing critical decisions such as research funding and promotions. Consequently, longer tenure at QU is associated with better Scopus representation. Furthermore, the *Global Top 2 percent of Highly Cited Researchers 2023* list, which uses Scopus data, includes 23 faculty members from QU Health Cluster (Ioannidis, 2023).

This research aligns with the global trend of increased scholarly output during COVID-19, with the highest publication counts observed between 2021 and 2023. The rise in research output may be attributed to remote work and virtual collaboration tools (McPhail et al., 2024). This publication increases highlights faculty adaptability to urgent global health concerns, necessitating rapid research dissemination during the pandemic. WoS indexed more faculty publications than Scopus after 2020, potentially due to a potential bias in WoS favouring the indexing of publications with international collaborations, which grew significantly during the pandemic (Boshoff & Akanmu, 2017; Carvalho et al., 2023; Cascajares et al., 2021). Additionally, faculty preference for publishing in open-access journals, supported by funding from Qatar University and Qatar National Library, further contributed to this trend since over 80% of COVID-19-related papers in 2020 were published in open-access journals, with 89% indexed in WoS and 83% in Scopus (Al-Abdulla & Dobreva, 2017; Qatar National Library, 2024; Teixeira da Silva et al., 2021). This likely reflects faculty efforts to maximise research visibility and accessibility through these funded publication avenues.

The discrepancy between CiteScore and impact factor is evident, with CiteScore being higher in 88% of shared journals. CiteScore’s broader scope of citations over a longer time frame often leads to higher values than the impact factor (Fernandez-Llimos, 2018; Lancho-Barrantes et al., 2010; Singh et al., 2021). While journal impact indicators signal a journal’s prestige, they may not always reflect the true quality or significance of research, particularly in specialised journals, which often have lower impact indicators (Casadevall & Fang, 2014; Mech et al., 2020; Oosthuizen & Fenton, 2014). The utilisation of field-weighted citation impact metrics, such as SJR2, facilitates effective cross-field comparison (Mech et al., 2020). This approach overcomes the limitations of traditional journal impact indicators in scientometric analyses (Mech et al., 2020; Oosthuizen & Fenton, 2014). This field-weighted approach for evaluating journal impact provides a more nuanced understanding of publication quality on a global scale, especially for specialised areas within pharmacy, compared to WoS and Scopus (Fernandez-Llimos, 2018).

The analysis revealed that 44% of shared journals had identical Q-ranking across WoS, Scopus, and SCImago. Scopus indexed more journals with higher Q-rankings due to its distinct evaluation criteria and recognition of pharmacy as an independent subject area (Elsevier, 2023; Feldner, 2024; Fernandez-Llimos, 2018). Differences in publication volumes and citation practices in indexed journals in each database may explain this variation, particularly since Scopus includes many open-access journals that attract higher citations due to wider accessibility (Feldner, 2024; Fernandez-Llimos, 2018). These factors contribute to higher Q-rankings for Scopus journals compared to WoS.

There was substantial overlap in subject area analysis across databases, predominantly related to pharmacy and its practice, health education, patient and medication safety, and chronic diseases management. This aligns with the findings of a 2022 scoping review of clinical pharmacy and practice research in 12 Arabic Middle Eastern countries, with Qatar identified as a major contributor, revealed eight prevalent themes in the field’s research output between 2009 and 2019 (Obaid et al., 2022). These themes correspond with the findings of this analysis, which highlighted subject areas covered by CPP faculty. The scoping review indicated that pharmacy education and professional development were the most frequent publication areas from Qatar (Obaid et al., 2022), while this analysis found that pharmacy practice and pharmacist services to be the most common. This discrepancy underscores the evolving nature of pharmacy research in Qatar, with a shift towards practice-oriented themes in recent years (Al-Worafi, 2023). This shift may reflect a growing emphasis on clinical pharmacy services and their impact on patient care within Qatar's healthcare system (Jebara et al., 2021).

The subject areas covered by faculty members align with the global research trends, as a 2019 study identified the core components of clinical pharmacy and practice research as medicines use, patient-centered care, and health services delivery (Hasan et al., 2019). The subject areas are distributed between theory-based research, focusing on pharmacies as subjects, and practice-based research, using pharmacies as sources of information (Koster et al., 2014). This alignment suggests Qatar's clinical pharmacy and practice research is on par with international developments, potentially influencing policy, education, and clinical practice both locally and internationally. Additionally, an increase from 38% to over 50% in regional and international collaborations at QU highlights the value of diverse perspectives in advancing scientific knowledge (Al Zaidan et al., 2022). The focus on practice-based themes may encourage more collaborative initiatives between academia and healthcare institutions locally and globally.

This scientometric analysis provides a comprehensive overview of the QU Clinical Pharmacy and Practice Department’s research productivity over 12 years, detailing publication trends, journal impacts, subject areas, and collaborations. It is important to note that this analysis is not intended to compare faculty members' productivity, as numerous factors influence publication output, including tenure at QU. Instead, the analysis aims to illustrate the department’s overall impact and research trends. The strengths of this analysis lie in its use of three major databases, each with distinct advantages, covering 12-year timeframe captures long-term research trends, while using multiple quantitative and qualitative indicators offers a robust assessment of research quantity, quality, impact, and benchmarking against regional and global trends. This multifaceted approach provides a better understanding of scientific contributions beyond mere publication counts. However, this scientometric analysis has some limitations. Focusing on a single department may limit the generalizability of the findings. Despite this, it provides valuable in-depth insights into the specific departmental research dynamics and practices, which can be applied to similar departments elsewhere. It is also important to acknowledge that database indexing and metadata errors or inconsistencies are common challenges in scientometric studies. These limitations were mitigated through rigorous quality control measures, including multiple rounds of data cleaning, verification, integrity checks, and the active involvement of a librarian experienced in databases and scientometric analyses to ensure accurate and reliable metadata retrieval, analysis, and interpretation. Future studies could explore how these findings apply to clinical pharmacy and practice departments in other institutions globally, without time constraints. Additionally, given the importance of research productivity indicators in decision-making for research opportunities, future studies should investigate the impact of funding and funding bodies on research output, particularly in relation to open-access journals.

## Conclusion

Scientometric analysis enables institutions to better align their research strategies with global scientific advancements and to identify growth opportunities. This 12-year scientometric analysis of Qatar University’s CPP department, based on data from WoS, Scopus, and Google Scholar, revealed steady growth in research output, with faculty members having active publication years for 85% of their tenures. The average annual publication rate was 5 articles per faculty member, with peak publication activity between 2021 and 2023. Most publications appeared in Q1 and Q2 journals, with a focus on pharmacy practice, health education, and medication safety. Google Scholar had the highest retrieval rate, though differences were noted across databases. The analysis highlights the department’s growth areas and opportunities for future development in clinical pharmacy and practice, offering insights for benchmarking against broader global pharmacy research trends.
